# Implementation of single IRB review for multisite human subjects research: Persistent challenges and possible solutions

**DOI:** 10.1017/cts.2023.517

**Published:** 2023-04-04

**Authors:** Jonathan M. Green, Polly Goodman, Aaron Kirby, Nichelle Cobb, Barbara E. Bierer

**Affiliations:** 1 Office of Human Subjects Research Protections, Office of Intramural Research, National Institutes of Health, Bethesda, MD, USA; 2 Harvard Catalyst, The Harvard Clinical & Translational Science Center, Harvard Medical School, Boston, MA, USA; 3 Association for the Accreditation of Human Research Protection Programs, Washington, DC, USA; 4 Multi-Regional Clinical Trials Center of Brigham and Women’s Hospital and Harvard, Boston, MA, USA; Division of Global Health Equity, Department of Medicine, Brigham and Women’s Hospital; Department of Medicine, Harvard Medical School, USA

**Keywords:** Single IRB, clinical trials, human subjects research, ethics review, institutional review boards

## Abstract

Revisions to the Common Rule and NIH policy require the use of a single Institutional Review Board (sIRB) for the review of most federally funded, multisite research, with the intent of streamlining the review process. However, since initial implementation in 2018, many IRBs and institutions continue to struggle with the logistics of implementing this requirement. In this paper, we report the findings of a workshop held in 2022 to examine why sIRB review remains problematic and propose possible solutions. Workshop participants identified several issues as major barriers, including new responsibilities for study teams, persistent duplicative review processes, the lack of harmonization of policies and practices across institutions, the absence of additional guidance from federal agencies, and the need for greater flexibility in policy requirements. Addressing these problems will require providing additional resources and training to research teams, the commitment of institutional leaders to harmonize practice, and policymakers to critically evaluate the requirement and provide flexibility in applicability.

## Introduction

In 2016, the National Institutes of Health (NIH) announced a new policy requiring the use of a single IRB (sIRB) for the review of most NIH-funded, multisite human subjects research [1]. The policy became effective in January 2018 and was soon followed by a similar mandate that was incorporated into the revised Common Rule (45 CFR 46.114). The primary rationale provided by both NIH and the Office of Human Research Protections (OHRP) to justify the sIRB requirement was to streamline the review process and eliminate inefficiencies inherent in a duplicative review process while not compromising human participant protections [1,2].

To facilitate the implementation of the sIRB requirement, in July 2016, the National Center for the Advancement of Translational Sciences (NCATS), a center within NIH, funded a collaborative effort to support the national adoption of sIRB review, termed Streamlined, Multisite, Accelerated Resources for Trials IRB Reliance Platform (SMART IRB) [3]. The SMART IRB platform provides a reliance agreement (the SMART IRB Reliance Agreement), a method for joining as a participating institution, resources, education, and a web-based process for participating institutions and their investigators to request, track, and document study-specific reliance arrangements. As of March 2023, more than 1080 entities are signatories to the SMART IRB Reliance Agreement.

It is now over 4 years since the implementation of the NIH sIRB policy and more than 2 years since the effective date of the Common Rule requirement. Yet, significant challenges with the implementation of sIRB remain [4–7]. The leadership of SMART IRB has organized an “Emerging Issues” annual workshop in which leaders in human participant protections gather to discuss sIRB review. The theme of the 2022 workshop was to identify “persistent barriers and future solutions to the success of single IRB.” Discussion topics were identified by workshop organizers based upon solicited input from human research protection program (HRPP) leaders throughout the country and from issues raised during discussions at prior SMART IRB talks and workshops. This paper summarizes the proceedings of the workshop.

## Workshop Organization

The workshop was conducted via video conferencing for one-half day in March 2022. Participation in the workshop was by invitation. In addition to 14 SMART IRB team members, 63 individuals attended, representing 47 institutions, funding agencies, an accrediting agency, government regulators, and independent IRBs.

After general opening remarks, workshop participants were organized into groups of 10–12 people and tasked with identifying the top issues related to the assigned focus areas for reviewing IRBs and relying institutions (see Table 1). Following 90 minutes of discussion framed around the discussion prompts, the workshop participants re-convened as a larger group to report their findings and engage in a broader discussion.

**Table 1.** Discussion topics and prompts provided to focus groups

Focus group	Discussion prompts
**Policy/Regulation**	Identify any specific issues and their possible solutions, related to provisions of the NIH sIRB policy or the revised Common Rule that hinder effective implementation of the sIRB requirement. Discuss how the lack of harmonization between NIH, HHS, and FDA impacts implementation of the sIRB requirement. Discuss whether the current exceptions to the policy requirements are sufficient, and if not, what other exceptions should be considered.
**Institutional Concerns**	Identify any institutional concerns that impact the acceptance and implementation of the sIRB requirements. For example, the impact on budget, planning, and allocation of resources.
**IRB Office Operational Challenges**	Identify any operational challenges within the IRB office of the reviewing IRB and/or the relying institution that continue to impact the ability to efficiently implement sIRB review.
**IRB Committee Reviewer Challenges**	Identify any challenges unique to sIRB review that are faced by IRB committee members when reviewing research conducted at sites for which they may have little or no familiarity.
**Harmonization of IRB Policies & Procedures**	Identify any barriers to the adoption of harmonized policies and operating procedures, such as those developed by SMART IRB, by participating institutions.

NIH, National Institutes of Health; IRB, Institutional Review Board; sIRB, single IRB; HHS, Health and Human Services; FDA, Food and Drug Administration; SMART IRB, Streamlined, Multisite, Accelerated Resources for Trials IRB Reliance Platform.

## Workshop Findings

### New Responsibilities for Study Teams

Virtually all workshop participants cited challenges faced by study teams in understanding and exercising the new responsibilities incurred by sIRB review as a significant impediment to the effective implementation of the sIRB requirement. This was true for both relying institutions as well as reviewing IRBs and reviewing IRB institutions, although the details differed depending on the role.

Study team responsibilities begin with recognizing the requirement for single IRB review and identifying a reviewing IRB to serve in that capacity. Many Principal Investigators (PIs) are either unaware of the requirement or mistakenly believe that their institutional IRB will automatically serve as the reviewing sIRB, leading to last-minute confusion, budgetary issues, and delay at the time of study start-up. Workshop participants thought that the revision of the requirement for providing an sIRB plan at the time of NIH grant submission to a “just-in-time” notification has contributed to this problem by allowing investigators to delay any planning for sIRB review.

Once an sIRB is selected, the lead PI and their research team often become the central point of contact between the reviewing IRB and the researchers at the relying institutions, responsible for coordinating communications between the relying PI, relying institution, and the reviewing IRB. The relying site staff are unlikely to be familiar with the reviewing IRB’s policies and procedures, and therefore the lead PI must ensure both communication and compliance. The complexity of this task and the resources needed to manage it effectively increase with the number of relying sites. Many research teams, particularly those conducting investigator-initiated research, are neither adequately resourced nor trained to manage this new work, resulting in ineffective communication, delayed and/or poor-quality submissions to the sIRB. This, in turn, leads to multiple cycles of clarification and response between the reviewing IRB, the lead study team, and relying sites, which can be time-intensive and frustrating for all parties.

Researchers at the relying institutions are often required to submit multiple applications, one to their own institutional IRB office (or other office within the HRPP that handles single IRB review) and one to the reviewing sIRB. The local submission can range from a simple request to rely on an external IRB to a more detailed and intensive submission that often increases the workload for the research team, especially when, as discussed below, the relying institution essentially requires a full duplicative IRB application.

### Relying Institution Reviews

An explicit intent of the sIRB model is that the relying institutions will not conduct their own IRB review of the study, ceding that responsibility to the reviewing sIRB. However, most relying institutions continue to require submission to their own institution for internal review, a review that can range from a relatively truncated “administrative review” to an extensive, in-depth process that, while perhaps not conducted by the convened local IRB, is essentially equivalent to full IRB review.

Relying institutions conduct internal review for several reasons. Importantly, although IRB review has been ceded, the institution remains responsible for the conduct of the research at its site and for “safeguarding the rights and welfare of human subjects,” a point made explicit in the revised Common Rule (45 CFR46.114(a)). The institution must therefore know what protocols are being conducted and are planned for at their site, including whether the proposed investigators are competent to conduct the research and whether the appropriate resources (e.g., nursing, pharmacy, imaging, technologies) are available.

Many studies undergo review by local institutional committees other than the IRB, termed ancillary review committees (e.g., institutional biosafety, radiation safety, nursing, pharmacy, conflict of interest) prior to the study being opened at a given site. Often, the electronic IRB submission system is used to manage these reviews, making separation of these processes challenging. An internal review is also necessary for the relying institution to determine the relevant “local context” information that must be provided to the reviewing IRB. Finally, institutions must know and report the volume and nature of the research for which it is responsible. The IRB office, HRPP, and institutional official rely on the submissions from their investigators to obtain these data for reporting purposes.

For all of the above reasons, submission of the proposed study for a local institutional review is generally the preferred (and often only) method to obtain the detail necessary to address these concerns. By conducting an internal review of ceded studies, institutions ensure compliance with their internal policy requirements and that the proposed human participant research can be conducted safely and responsibly at their site. In institutions with their own internal IRB, the IRB office is often the operational arm of the HRPP that is typically charged with coordinating these activities, ensuring that appropriate internal review has occurred, and assuring that required institutional approvals are in place prior to the initiation of research.

While some review by relying institutions is necessary, workshop participants felt strongly that many institutions conduct unnecessarily detailed duplicative reviews. For example, in addition to reviewing for local policy requirements, some institutions perform a complete assessment of the regulatory criteria for approval, while others may review for the applicable subparts, such as when minors are included in the research. Local review for regulatory requirements runs counter to the intent of utilizing an sIRB and abrogates any potential efficiency gains. Alternate systems are not in place to obviate duplicate submission and yet achieve institutional goals. Greater clarity in the roles and responsibilities of relying institutions and reviewing IRBs, as well as tools to help institutions streamline their internal review processes and improve communications and information exchange, are needed to mitigate this problem.

### Lack of Harmonization of Processes Across Reviewing IRBs

IRB offices have developed different internal processes that serve their institutional needs. While there are similarities, each IRB and HRPP have evolved their own set of operational business processes. Differences range from important policy requirements, such as the time frames and thresholds for reporting concerns to the IRB, to the less consequential details of IRB electronic submission systems, forms, and data collection formats.

As is the case for research teams, IRB offices are affected by the lack of harmonization of policies and practices. Relying institutions must learn the requirements of each reviewing IRB to allow for review to proceed. Conversely, the reviewing IRB must learn and understand how each relying site provides oversight of their investigators and research staff in order to execute their responsibilities and assure a safe environment for the conduct of human participant research.

These differences result in all parties constantly learning and adapting to unfamiliar processes, which affects not only the efficiency of sIRB review but also administrative burden and compliance concerns. Workshop participants advocated strongly for harmonization and simplification of processes, reducing wasted time and effort, and potentially improving compliance. A commitment to change local customs and invest in alignment in both operations and technologies would arguably result in long-term cooperative system solutions and efficiencies. A standing committee within SMART IRB, the Harmonization Steering Committee, is charged with identifying areas in which adoption of uniform practices is feasible and likely to yield meaningful gains in efficiencies. Despite developing recommendations for standardizing processes across HRPPs, organizations appear to be reluctant to change and adopt new business processes.

### Need for Cross-regulatory Guidance on Applicability of sIRB Requirement

Workshop participants stressed the need for additional guidance from the NIH and the Office of Human Research Protections (OHRP). There are several differences between the NIH policy and the Common Rule requirement that have led to confusion within institutions. For example, the Common Rule requirement applies to “cooperative research” whereas the NIH policy refers to “multi-site studies where each site will conduct the same protocol.” To illustrate, workshop participants indicated that a study in which each site conducts distinct activities as part of a single project (e.g., one site does only imaging, another only biospecimen analysis, another enrolls participants), might be considered cooperative research subject to the Common Rule requirement yet not fall under the scope of the NIH policy. Moreover, the exceptions to the requirement for single IRB review issued by NIH and OHRP do not align, adding to the confusion as institutions try to comply. For example, NIH provides categorical exceptions and has the ability to provide exceptions for individual studies on a case by case basis, whereas OHRP issues only categorical exceptions.

Further, studies may fall under the oversight of multiple regulatory bodies and agencies. An NIH-funded investigator-initiated study of an investigational medical product would have to comply with NIH policy, the Common Rule, and applicable FDA regulations. While at this time the FDA has not adopted an sIRB requirement, a recent notice of proposed rulemaking (NPRM) signals their intent to align [8]. While the final rule may change, the NPRM suggests that the forthcoming FDA requirement will differ from both the NIH and Common Rule sIRB requirements. For example, if adopted as proposed in the NPRM, the FDA sIRB requirement will not apply to research that is not conducted under an Investigational New Drug (IND) application or for device studies subject only to the abbreviated Investigational Device Exemption (IDE) requirements. However, those same studies may be subject to the Common Rule and/or NIH sIRB requirements. Guidance is needed as to how these policies work together and what institutions are to do when there appear to be conflicting requirements.

### Need for Greater Flexibility and Exceptions

Both the NIH policy and revised Common Rule requirement apply broadly to nonexempt human participant research with few exceptions, making no distinction based on the nature of the research or its inherent risks. Workshop participants thought greater flexibility in the application of the sIRB requirement is needed so that when the sIRB requirement applies it can be expected to increase efficiency or reduce burden. For example, research that qualifies for expedited review is typically approved with a turnaround time of under 2 weeks at most institutions [9]. These studies are minimal risk and generally straightforward and may even qualify for waiver of informed consent, hence there is little need for changes to the study and/or consent document. For studies such as these, which in most cases no longer require continuing review under the revised Common Rule, the additional burden on study teams and the relying institutions often exceeds that needed to obtain approval from each institutional IRB.

Workgroup participants advocated for consideration of additional exceptions from the sIRB requirement, including when participating sites are conducting different aspects of the research in a multisite project. For example, one site may be enrolling participants to administer an investigational drug, while a second site may only be imaging the participants, while a third site is analyzing identifiable biospecimens and data. Local IRB review at each site might be preferable as the activities are substantially different between the sites, including one that is conducting only minimal risk research activities.

A third consideration for flexibility and/or exceptions occurs when there is the potential for significant differences in local context or state laws that may apply to the research. For example, in comparative effectiveness research, any significant practice variation across sites will impact the risk and benefit assessment, rendering knowledge of local practice essential for appropriate IRB review. The sIRB would be unlikely to possess this information. A second example is FDA-regulated emergency research conducted with an exception to informed consent. These studies require extensive local community stakeholder engagement, a process in which the IRB is encouraged to play an active role. A single central IRB is unlikely to be able to engage effectively with multiple, geographically dispersed communities.

### Non-compliance

The review of noncompliance in human participant research was raised as one of the most challenging issues facing institutions, particularly for those relying on an sIRB. Differences in the definition of noncompliance, when that noncompliance is considered serious or continuing, appropriate corrective actions, and oversight of the corrective actions lead to situations in which the same event can have widely disparate consequences depending on the reviewing IRBs policies, practice, and experience. Further, the reviewing IRB’s authority is limited to taking actions on a study, not an individual investigator; authority for the investigator remains with the investigator’s home institution, which must manage the potential noncompliance based on determinations of an outside body and potentially without complete or adequate information.

Other operational challenges were discussed including confidentiality protections during the investigation of potential noncompliance, poor communication between the entities including instances of nonresponsiveness by the relying institution to queries from the sIRB, and concerns over reporting responsibilities to federal agencies, among others. Workshop participants suggested that harmonized approaches to the identification, investigation, review, communication, and reporting of instances of potential noncompliance would be beneficial.

### Local Context

A reviewing IRB is required to consider any factors that might impact the approvability of a study at a particular site. These include a wide variety of issues broadly referred to as “local context” considerations. Examples include local or state laws that affect some sites but not others, local clinical practice variation in the delivery of medical care, or unique aspects of the population of individuals that might be expected to enroll at a particular site.

Reviewing IRBs solicit input from the relying sites on local context considerations. However, there is little uniformity in what information is requested, what information is provided, and how it is communicated. Reviewing IRBs may fail to request, and relying sites may fail to include needed information. The consequences of a reviewing IRB having incomplete or inadequate local context information can be significant and risk rendering the adequacy of the sIRB determination suspect. For example, the IRB may approve research that runs counter to state law or not take into consideration variations in potential risks to subjects created by state law, or fail to realize that due to regional practice variation a clinical intervention as “standard of care” at one site is, in fact, nonstandard at others.

### Solutions

Approaches identified by workshop participants to address, improve, and/or solve the problems described above can be summarized in three categories: resources, harmonization, and policy flexibility.

### Resources

Investigators and their study teams are responsible for and must be competent to conduct the research, but in most cases, they are neither trained nor resourced to handle the administrative work of coordinating multisite IRB submissions. Workshop participants suggested that institutions are advised to provide dedicated support and infrastructure to assume these new responsibilities. Specialized staff could function as the central liaison between study teams and reviewing IRBs and serve a number of study teams, thereby acquiring a core knowledge base and expertise of processes and policies to manage IRB processes efficiently. Communication between relying institutions and reviewing IRBs would be facilitated as the teams develop knowledge of institutional processes and relationships with stakeholders. These specialized staff could also contribute to developing common processes, help with change management, and participate in nationally coordinated efforts to harmonize practice. Developing centralized support staff is likely to be both more efficient and effective than relying on training individual study teams. Each study team is likely to be an infrequent user and as such will not develop expertise or retain training. Furthermore, a strategy of targeting study teams means many such teams will need to be trained (and re-trained) instead of a focused approach targeting centralized support staff.

In addition to human resources, workshop participants indicated that technological improvements would alleviate many of the current frustrations [10]. An interoperable, workflow-based, electronic IRB management system available to all institutions would simplify communications and document flow and obviate the need for duplicative submission to the relying institution. Data relevant to the institution or to the specific protocol would be instantly available, current, detailed, and downloadable. Further, a common system would propel harmonization of processes, as the process itself would be embedded in the workflow of the IT solution.

### Policy Flexibility

The current regulatory and policy requirements for sIRB review are inclusive of almost all clinical research subject to IRB review, with only limited exceptions. Given the extensive community experience, workshop participants recommended that a rigorous examination to determine when sIRB review meets the policy objectives of increasing efficiency and reducing burden should be undertaken, and the agencies are requested to consider creating exceptions in those situations when it does not.

### Harmonization

Harmonization of policies and practices across IRBs would greatly increase the efficiency of sIRB review. While all US-based IRBs are governed by the same sets of regulations, workshop participants indicated that each has interpreted the same regulatory requirements differently resulting in substantial policy variation. For example, the requirement for prompt reporting of an unanticipated problem may mean 24 hours at institution A, five business days at institution B, and ten calendar days at institution C. As another example, each uses their own customized IRB application, soliciting what is essentially the same information but asking for it in different ways with different questions, for unclear benefit.

SMART IRB has been working to address this problem through its Harmonization Steering Committee (HSC). The HSC consists of HRPP leaders from across the country and identifies areas in which harmonization of policy and practice would be beneficial to sIRB review. Workgroups create guidance and tools for institutions that if adopted would harmonize and streamline operations. However, despite an abundance of resources now being available [11], many institutions remain reluctant to change, limiting the impact of these efforts. The reasons why institutions have not harmonized policies and practices are varied. For some, it is simple inertia; for others, it may be a lack of the needed institutional support to drive change as well as limited or no funding to develop and implement new processes. While regulations can mandate the use of an sIRB, they cannot mandate processes. Unless institutions begin to experience consequences, such as the inability to participate in multisite research, there may be little reason for many institutions to change. However, forums that bring together IRB and HRPP thought leaders, like the SMART IRB Emerging Issues workshop, appear to be one way to encourage consideration of the need to harmonize and build consensus on processes and policies.

At a time when most research was single site, perhaps the differences in institutional processes were of little consequence. However, in the current era of team science and large multisite clinical research projects involving numerous institutions, these differences lead to confusion and inconsistencies and are no longer defensible. The ultimate goal of HRPPs and their associated IRBs do not fundamentally differ between research sites. While there may be many ways to accomplish these goals, in the interest of science and evidence-based improvements to human health, it is time we all agreed on one approach.

## Conclusion

Whether or not causative, redundant IRB reviews at each site in a multisite clinical trial have long been held responsible for delaying study start-up; eliminating duplicative review was one of the primary intents of the NIH and revised Common Rule requirements for use of an sIRB. However, many of the anticipated benefits of the policy have yet to be realized. Investigators and HRPP offices express frustration with the requirement, and many feel that it has shifted or even increased burden rather than reduced it. Resources and systems are not yet in place to test whether time to study start-up is routinely decreased or to track appropriate metrics, although anecdotal reports suggest that this is possible [12].

We believe that the goal of sIRB review to enhance efficiency and reduce burden without adversely impacting human participant protections remains achievable. However, without additional work and institutional commitment, this goal is unlikely to be realized. Workshop participants suggested that institutions must commit resources to support their investigators and study teams with their new responsibilities. HRPP leadership must be willing to compromise and change policy and practice to achieve the needed levels of harmonization. IT infrastructure should be built to be interoperable, available, and workflow-based, with appropriate security to protect intellectual property, institutions, investigators and their study teams, and, importantly, participants. Finally, policymakers should critically evaluate the requirement for sIRB review to determine the circumstances under which it achieves its intended effect and, alternatively, when it is more likely to add burden rather than reduce it, adjusting the policy scope accordingly.
